# The impact of aerobics on mental health and stress levels: A visualization analysis of the CiteSpace map

**DOI:** 10.1371/journal.pone.0300677

**Published:** 2024-03-19

**Authors:** Jianxin Zhao, Yabing Bai, Yongjing Yang, Xiaolei Li

**Affiliations:** 1 Department of Physical Education and Teaching, Hebei Finance University, Baoding, Hebei, China; 2 School of Accounting and Finance, Changsha Commerce & Tourism College, Changsha, Hunan, China; Hosei University: Hosei Daigaku, JAPAN

## Abstract

This study aims to integrate research in the field of aerobics and mental health through the visualization analysis method of the CiteSpace map, to clarify the impact of aerobics on mental health and stress levels. Firstly, based on the literature method, pieces of literature related to aerobics and mental health are searched and collected. Secondly, the visualization analysis method of the CiteSpace map is employed to summarize and analyze the contents of the literature, involving statistical analysis of the annual number of publications, analysis of author characteristics, and analysis of publishing institution characteristics. In addition, keyword co-occurrence analysis and keyword cluster analysis are also conducted in related research fields. Among them, the Log-Likelihood Ratio is used in keyword cluster analysis. Finally, the results are analyzed using the visualization analysis method of the CiteSpace map and the statistics-based comprehensive results. The results demonstrate that in the recent 20 years, the average annual number of articles in related fields exceeds 190. The high-yield authors are distributed in economically developed areas, and the cooperation among authors is scattered. In the keyword clustering results, a total of 77 cluster labels are obtained. The Q value of the clustering module is 0.89, and the average clustering profile silhouette (S) value is 0.92, indicating that the clustering structure is significant and the results are reasonable. The aerobics cluster contains the most closely related keywords, covering mental health and stress levels. Data analysis based on existing studies reveals that aerobics has a significant impact on mental health and stress levels. Individuals participating in aerobics show obvious improvement in mental health inventory (MHI) scores (t(100) = 4.32, p<0.05). Individuals participating in aerobics present a remarkable reduction in the questionnaire evaluation of stress levels (t(100) = -3.91, p<0.05). This study’s results support aerobics’ positive effects on mental health and stress levels.

## Introduction

With the fast pace of life and the increasing pressure of competition in modern society, mental health problems are increasingly becoming the focus of attention [1]. Individual mental health impacts happiness and quality of life and is key to social stability and development [2]. However, many factors in modern life, such as work pressure, interpersonal tension, academic burden, etc., often lead to the emergence and aggravation of individual mental health problems. Therefore, finding effective methods and interventions to improve mental health and reduce stress levels is of great practical significance and application value. Currently, mental health research mainly focuses on personality levels and stress relief methods. For example, Huang et al. (2023) achieved remarkable results in studying mental health at the personality level [3]. Aerobics, as a comprehensive movement integrating exercise, music, and dance, has attracted much attention among the many ways of mental health intervention. Participating in aerobics exercises can promote the operation of the body’s metabolism and circulatory system, enhance muscle strength and flexibility, and also help release pressure and relieve anxiety [4, 5].

Studies have also shown that participating in aerobics can improve mental health [6, 7]. Aerobics exercises can reduce anxiety levels and alleviate depressive symptoms and improve self-esteem and self-confidence [8]. However, some studies have disagreed or failed to reach a consensus [9]. Previous studies mainly described aerobics’ movement forms, training methods, and exercise effects. Still, little was known about its impact on mental health and stress levels [10]. Moreover, there are some differences and inconsistencies in the conclusions of existing studies [11]. Hence, it is necessary to conduct a deeper exploration and comprehensive analysis of the relationship between aerobics exercise and mental health, to clarify its impact on mental health and stress levels.

The objective of this study is to comprehensively analyze the research on aerobics exercise and mental health, and use the visualization analysis method of the CiteSpace map to clarify the influence of aerobics exercise on individual mental health and stress level, and explore its potential mechanism. This study uses the literature method to search and collect the literature related to aerobics exercise and mental health and employs the visualization analysis method of the CiteSpace map to summarize and analyze the content of this literature. Among them, keyword co-occurrence analysis and keyword cluster analysis will help to reveal the association and influence mechanism between aerobics exercise and mental health. Meanwhile, data analysis (DA) based on existing research is conducted to quantify and interpret the effects of aerobics exercise on individual mental health and stress levels. This study’s development is expected to provide a scientific basis for further research and practice in related fields.

## Material and methods

### Literature review

Studies in recent years have shown its positive effects on aerobics’ impact on mental health. Many studies focused on the influence of aerobics on anxiety and depression, showing significant effects [12]. For example, Farkas et al. (2023) found that aerobics can reduce anxiety levels and improve emotional states [13]. Similarly, Jain et al. (2022) showed that aerobics training could help reduce depressive symptoms and improve quality of life [14]. These findings highlighted the potential of aerobics as a form of mental health promotion. In addition, many studies presented that aerobics positively impacted an individual’s self-esteem and self-confidence. Monz et al. (2021) believed that participating in aerobics could enhance individuals’ positive evaluation of themselves, which was of great significance for mental health and social adaptability. Studies also found that aerobics could effectively reduce stress levels and improve emotional state and coping ability [15]. Gonçalves et al. (2022) pointed out that individuals who participated in aerobics showed significant improvement in stress levels [16]. Carreira Míguez and Clemente Suárez (2023) found that individuals who participated in aerobics experienced significant stress reduction after exercise, which was more remarkable in long-term exercise [17]. Mahindru et al. (2023) proposed that after participating in aerobics training, individuals had higher happiness and satisfaction, closely related to improving their mental health [18]. Besides, they found that aerobics might help increase an individual’s self-awareness and motivation. Looser et al. (2023) demonstrated that aerobics could improve individuals’ positive evaluation of their image, thus improving their mental health [19]. Sabri et al. (2023) exhibited that aerobics exercise could improve self-esteem [20].

Despite these positive findings, there was still some controversy and uncertainty about aerobics’ specific mechanisms and effects on mental health [21]. The results of the studies were inconsistent, possibly because of differences in research methods, sample characteristics, and other interfering factors. Some studies were limited to observational design and lacked the rigorous design of randomized controlled trials. Hence, the explanation of causality needs further research [22]. In terms of methods, existing researches mainly use questionnaire survey, experimental design, and observational research to collect and analyze data [23]. These methods provide valuable information in uncovering the effects of aerobics on mental health, but more diversity and in-depth study design are still needed to support their conclusions.

To sum up, although some studies revealed that aerobics had a positive impact on mental health, the understanding of its specific effects and mechanisms still need to be further explored. Based on the visualization analysis method of the CiteSpace map, this study synthesized the research in aerobics and mental health, aiming to clarify the influence of aerobics on mental health and stress levels. Through systematic review and DA, this study offered a new perspective and in-depth understanding of the research in this field and provided a scientific basis for applying aerobics in promoting mental health.

### Data collection method

**Fig 1:**
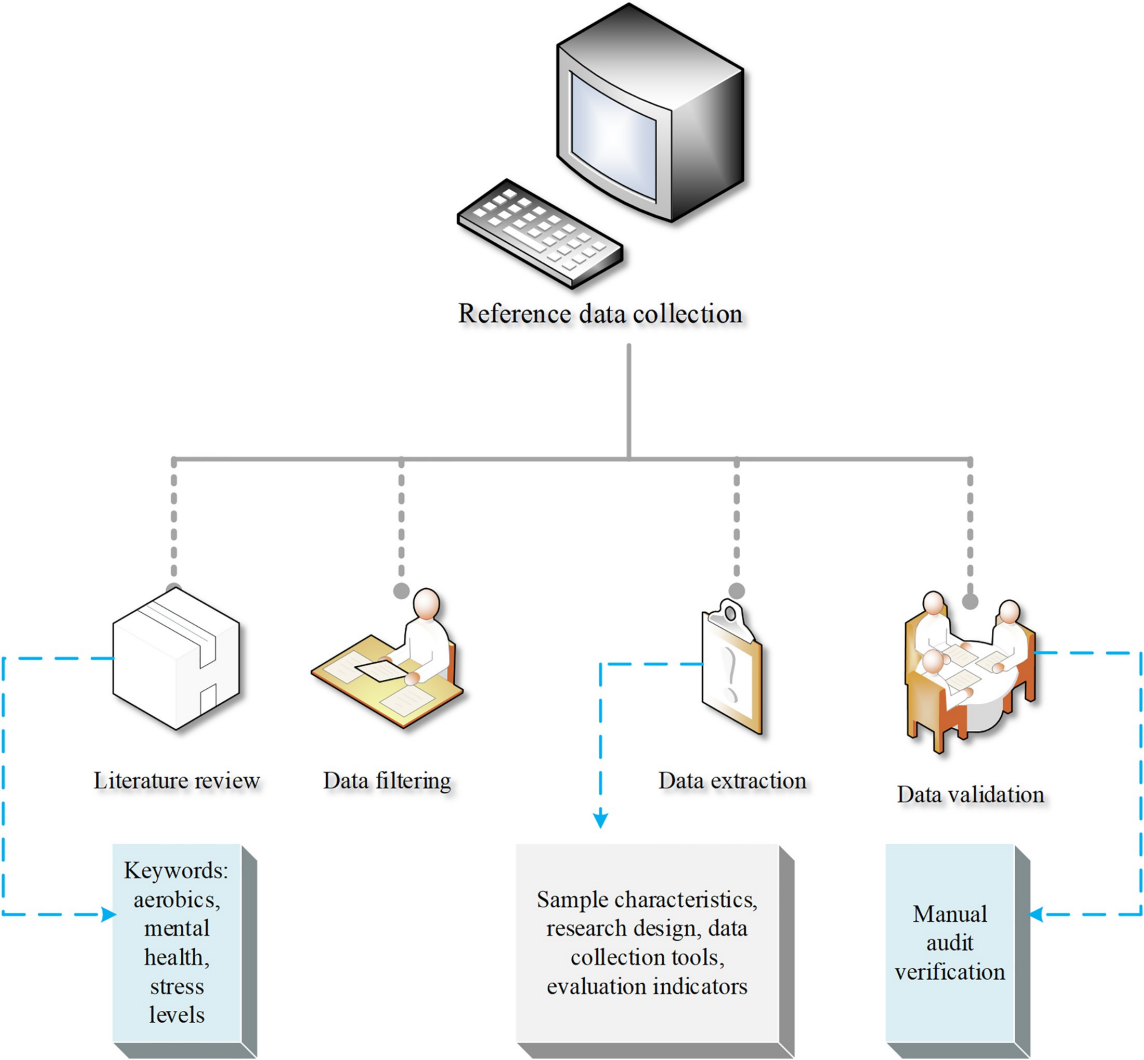
Data collection methods and steps.

The following data collection methods are used in this study to obtain relevant information to support the analysis of the effects of aerobics on mental health and stress levels. Specific data collection methods and steps are displayed in Fig 1.

In Fig 1, a literature review is conducted first. Research literature related to aerobics and mental health is collected through a search of academic databases and journal websites [24]. Second, according to the research literature obtained in the literature review, the data is systematically acquired and sorted out.

Strict standards and methods are employed in the data collection process to ensure the reliability and accuracy of the data. In this process, statistical methods are mainly employed. The studies use a variety of statistical techniques to analyze the data and draw conclusions. Only these studies are included to ensure that the data collected in the study are scientifically reliable and explanatory. Specifically, various statistical data involved here are collected, including descriptive statistical analysis, T-test, and correlation analysis. The calculation involved is demonstrated in Eqs (1)–(4) [25].


μ=(ΣX)/n (1)

Mean (*μ*) and standard deviation (*σ*) are commonly used descriptive statistics, ΣX represents the sum of the individual observations in the sample, and n refers to the size of the sample.


$$\sigma =\sqrt{\left(\Sigma (x-\mu {)}^{2}/(n-1)\right)}$$ (2)

Eq (2) is the calculation of standard deviation.


$$r=\Sigma ((X-\mu X)(Y-\mu Y))/\sqrt{\left(\Sigma (X-\mu X{)}^{2}\Sigma (Y-\mu Y{)}^{2}\right)}$$ (3)

Eq (3) is the commonly used calculation representation of Pearson’s correlation coefficient. Among them, *X* and *Y* represent the values of the two variables; *μX* and *μY* indicate the means of the corresponding variables, respectively.


$$t=\left(X_{1}-X_{2}\right)/\sqrt{\left(\left({s_{1}}^{2}/n_{1}\right)+\left({s_{2}}^{2}/n_{2}\right)\right)}$$ (4)

Eq (4) is the calculation of the T-test. *X*_*1*_ and *X*_*2*_ represent the mean of the two groups of samples; *s*_1_ and *s*_2_ signify the standard deviation of the two groups of samples; *n*_1_ and *n*_2_ refer to the size of the two groups of samples, respectively.

### The visualization analysis method of the CiteSpace map

**Fig 2:**
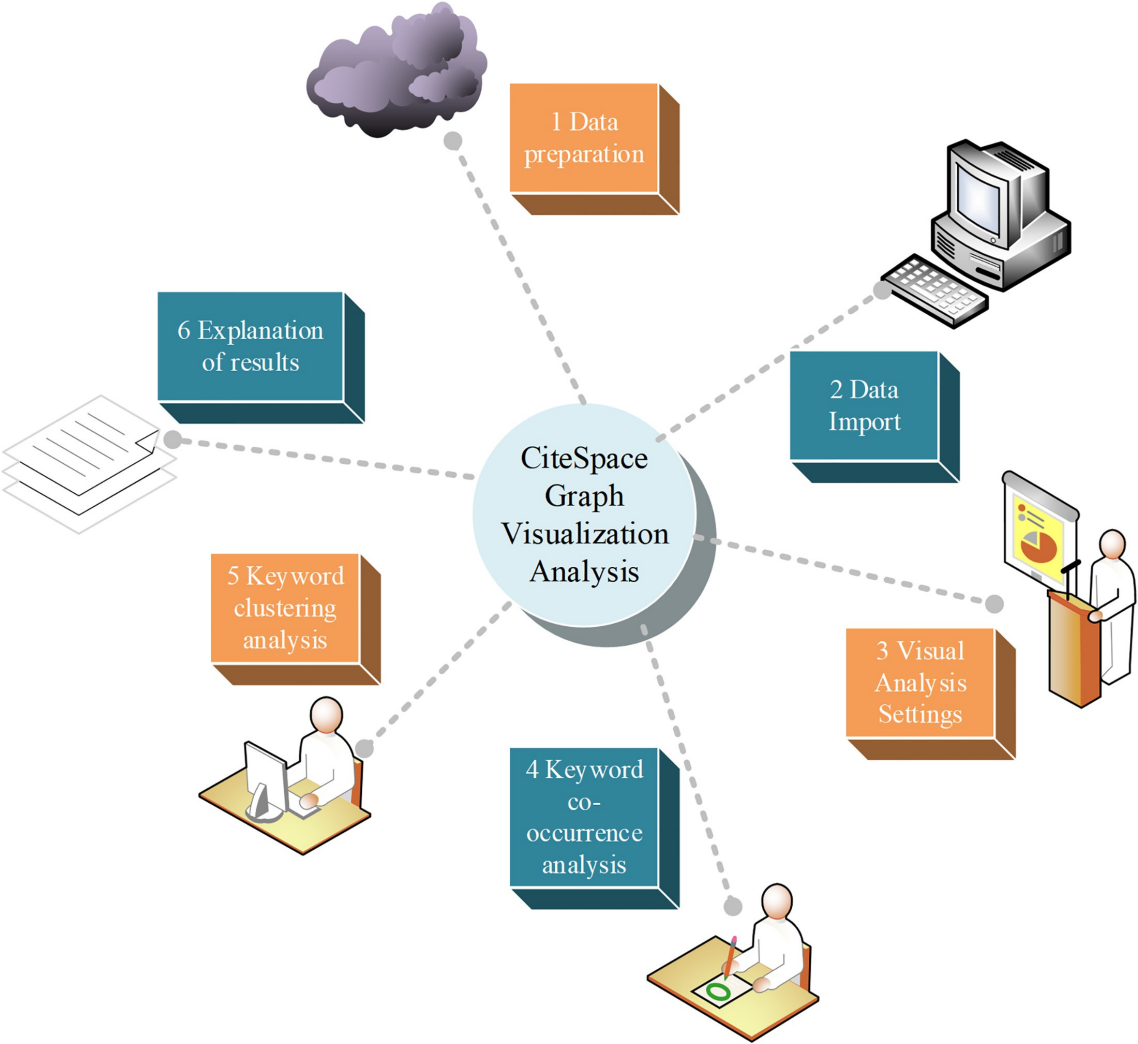
The visualization analysis steps of the CiteSpace map.

The CiteSpace is a visualization-based analysis tool that is widely used in bibliometrics and scientific research [26]. It can help researchers find correlations, trends and development patterns among literature, and extract useful information from large amounts of literature data. the visualization analysis method of the CiteSpace map is used in this study to delves into the effects of aerobics on mental health and stress levels. The CiteSpace map mainly consists of the following steps, as presented in Fig 2 [27].

Based on the content in Fig 2, it is necessary to collect research literature related to aerobics and mental health in data preparation, and sort and prepare these pieces of literature for subsequent import into CiteSpace software for analysis. After the literature collection is completed, the literature is organized into a format suitable for importing CiteSpace software. The accuracy and reliability of subsequent analysis can be improved by accurately sorting and labeling literature data.

In the data preparation phase, code can be utilized to assist the literature collection process. The code can help automate the search of academic databases, journals, and conference papers, and access literature information related to aerobics and mental health. Python and Scholarly libraries are used to search academic documents and obtain codes, as exhibited in Table 1 [28].

**Table 1:** Literature search code.

No.	Import scholarly
**1**	# Define search keywords
	keywords = ["aerobics", "mental health", "stress level"]
**2**	# Create an empty literature list
	articles = []
**3**	# Search for each keyword
	for a keyword in keywords:
	# Search and obtain relevant literature results
	search_results = scholarly.search_pubs(keyword)
	# Iterate through the search results and add the literature information to the list
	for result in search_results:
	article = {
	"Title": result.bib.get("title", ""),
	"Author": result.bib.get("author", ""),
	"Abstract": result.bib.get("abstract", ""),
	"Keywords": result.bib.get("keywords", []),
	"Citations": result.citedby
	}
	articles.append(article)
**4**	# Print literature list
	for an article in articles:
	print("title:", article["title "])
	print("author:", article["author "])
	print("abstract:", article["abstract "])
	print("keywords:", ", ".join(article["keywords"]))
	print("citations:", article["citations"])
	print("------")

In CiteSpace, parameters are set, including time range, node size, and color settings. These settings affect the presentation and readability of the map. The set time was chosen from 2000 to 2022 to cover relevant studies over the last 20 years. Due to the increased attention to health and lifestyle after 2000, and the increased frequency of studies on physical activities such as aerobics, the year 2000 was chosen here as the beginning of the search. Depending on the citation frequency, the node size is set. Citation frequency reflects the number of times a literature has been cited in other studies and can be used to measure its importance and impact. The node’s color is set according to the keywords’ different themes. The specific parameter settings are outlined in Table 2 [29].

**Table 2:** Parameter setting.

Item	Time		Node			Connection strength	Does the network use cropping
	Range	Segment	Type	Size	Threshold value		
**Specific setting**	2000–2022	1-year	Author	Citation frequency	Top50	Default value	Not used

The visualization analysis method of the CiteSpace map provides an intuitive and comprehensive framework to help researchers gain insight into the impact of aerobics on mental health and stress levels. It can find the correlation among works of literature, research hotspots, and trends, and offer valuable guidance and reference for further research. This study uses the CiteSpace map to explore and explain the research in the field of aerobics and mental health.

### Keyword analysis method

In keyword co-occurrence analysis, first, it is necessary to import the collected relevant research literature into CiteSpace software and extract keyword information from the literature. Next, a threshold needs to be set to filter out high-frequency keywords. The selection of threshold values can be based on statistical methods, which are usually sorted according to the keyword occurrence frequency. The top keywords are selected as high-frequency keywords [30]. In a network diagram, each keyword represents a node, and edges connect the co-occurrence relationships between keywords. Co-occurrence frequency can be determined by counting the number of times two keywords appear in the same literature. If two keywords appear more often at the same time in multiple literatures, their co-occurrence frequency is higher, and vice versa, it is lower [31].

The co-occurrence frequency between two keywords can be calculated using Eq (5) [32].


Co(A,B)=Σn(A,B)/(Σn(A)*Σn(B)) (5)

Σ*n*(*A*, *B*) represents the sum of the co-occurrence times of keywords A and B in all literature; Σ*n*(*B*) and Σ*n*(*A*) refer to the total number of occurrences of keywords B and A in all literature.

A common method in keyword cluster analysis is based on a modularity maximization algorithm, which can be used to divide keywords into different clusters. Assuming there are *N* keywords, it is necessary to construct a keyword co-occurrence matrix C of *N*×*N*, where *C*[*i*,*j*] represents the co-occurrence times of keywords *i* and *j*. Then an N-dimensional vector s is defined, where *s*[*i*] means the number of the cluster group to which the keyword *i* belongs. The calculation for modular Q is as follows [33].

$$Q=(1/(2m))\sum {\left[C[i,j]-\left(k[i{]}^{⋆}k[j]/(2m)\right)\right]}^{⋆}\delta (s[i],s[j])$$ (6)

*m* refers to the total number of the keyword co-occurrence times; *k*[*i*] represents the degree of the keyword *i*, that is, the sum of the number of cooccurrences of the keyword *I*; δ stands for the Krodecker function, which is 1 when *s*[*i*] equals *s*[*j*], otherwise, it is 0.

The algorithm’s goal is to maximize the modularity Q by assigning keywords to different clustering groups so that the modularity reaches the maximum. This can be achieved through iterative optimization algorithms, where each iteration reassigns keywords to clustering groups for higher modularity.

This study utilizes the Log Likelihood Ratio (LLR) for keyword cluster analysis. The LLR is a clustering algorithm based on probability and statistics that measure the correlation and similarity between keywords [34]. It calculates the LLR between keywords based on their co-occurrence frequency in the literature, to determine whether they have a significant correlation. The larger the LLR, the higher the correlation between keywords. Its main advantage is its accuracy in measuring correlation and similarity between keywords. This algorithm calculates the co-occurrence frequency of keywords in the literature, and then evaluates the LLR between keywords to determine their correlation. This method not only considers the frequency of keywords, but also comprehensively considers their occurrence in different literatures, to more accurately reflect the relationship between keywords. Compared with simple co-occurrence frequency calculation methods, the log-likelihood algorithm can exclude some possible random co-occurrence cases and accurately capture the correlation between keywords. Compared with other clustering methods, the proposed algorithm is more accurate and precise in dealing with the correlation between keywords. Some traditional methods for calculating co-occurrence frequency may be limited by the number of keyword occurrences in the literature and cannot fully consider the subtle differences between keywords. The log-likelihood algorithm overcomes this problem by introducing a probability model into the statistical calculation to reflect the relationship between keywords better.

When conducting keyword cluster analysis, a commonly used indicator is the LLR, which evaluates the degree of correlation between two keywords in the same cluster. The calculation for the LLR is illustrated in Eq (7).


$$\begin{array}{c} \text{LLR}=2^{*}\left(n11^{*}log\left(n11^{*}N\right)+n12^{*}log\left(n12^{*}N\right)+n21^{*}log\left(n21^{*}N\right)+\right.\\ \left.n22^{*}log\left(n22^{*}N\right)-\left(n1^{*}log\left(n1^{*}N\right)+n2^{*}log(n2*N)+n^{*}log(n*N)\right)\right) \end{array}$$ (7)

*N* and *n* = *n*1+*n*2 represent the total number of occurrences of the keyword and two keywords; *n*1 = *n*11+*n*12 and *n*2 = *n*11+*n*21 indicate the number of occurrences of the keywords *i* and *j*; *n* = *n*1+*n*2 indicates the total number of occurrences of the two keywords.

## Results and discussion

### Results of literature comprehensive analysis

In the process of literature collection in this study, it is found that the overall number of literature related to aerobics, mental health, stress level, and map visualization shows an upward trend, with an average annual number of publications of about 190 articles, which can be roughly divided into three stages, as plotted in Table 3.

**Table 3:** Literature collection results.

Group	Year	Number of publications
**1**	2000–2009	Stable development
**2**	2010–2017	Unstable fluctuations
**3**	2018–2022	Stable development

**Fig 3:**
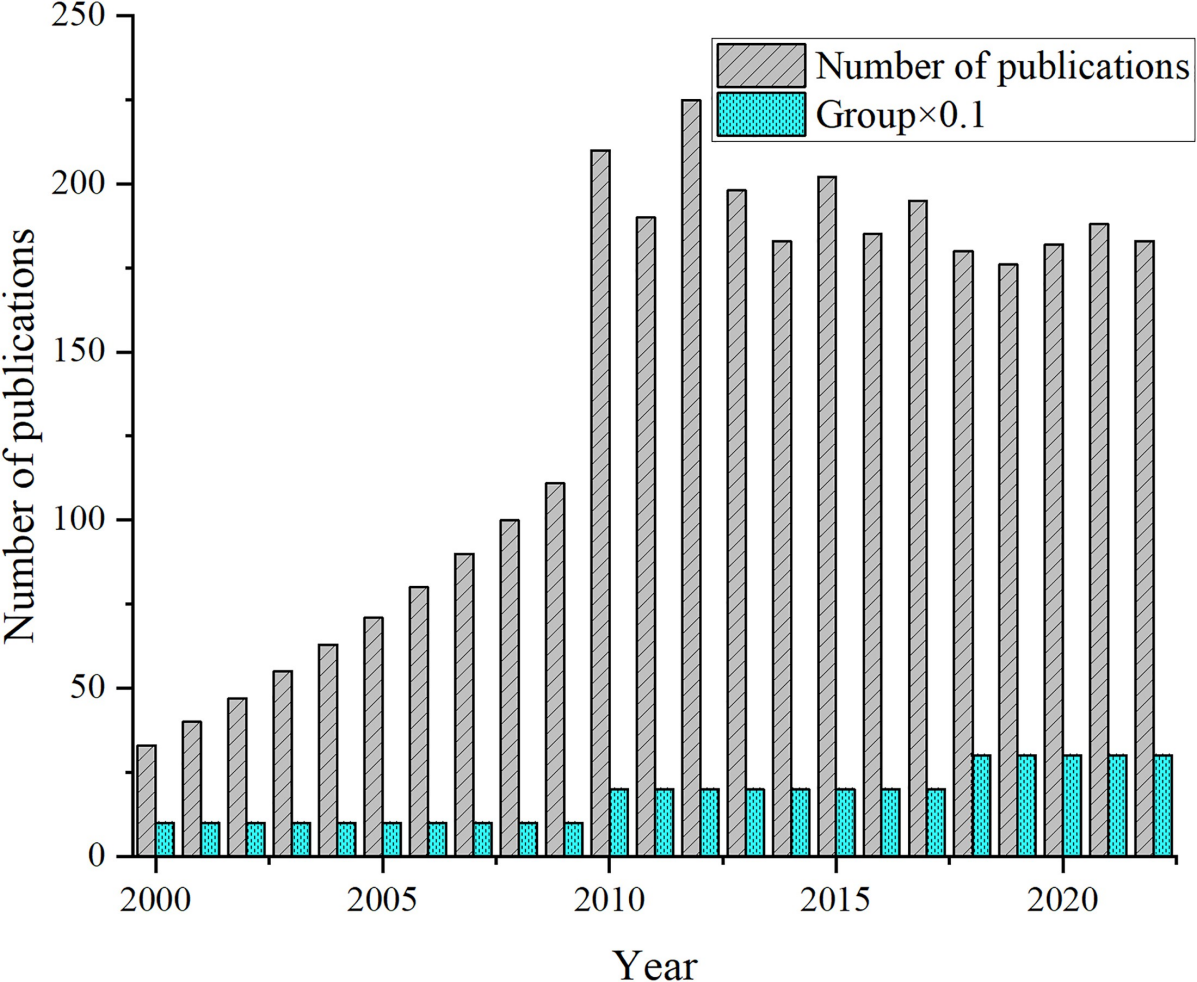
Literature collection results.

As shown in the bar chart of Fig 3. For this effect, the number of groups in Fig 3 is multiplied by 0.1.

Fig 3 demonstrates that the first stage is between 2000 and 2009, with an annual number of publications increasing from 33 in 2000 to 278 in 2009. The second stage is from 2010 to 2017, during which the annual number of publications fluctuates, and the overall is unstable, with an average annual number of approximately 200 articles. The third stage is from 2018 to 2022, with several publications of about 175 articles in related fields, and the overall exhibit a stable development trend. In summary, scholars have maintained a sustained focus on literature related to aerobics, mental health, stress levels, and map visualization, and research in this field may continue to develop steadily in the future. In a further analysis of trends in the number of publications in research journals, the study noted a significant increase in the number of publications in 2010. This may have something to do with some new journals coming into use. Therefore, this study further explores this phenomenon and proposes possible explanations based on a literature review and academic trends. First, the sudden increase in publications in 2010 reflects the development and changes in the academic community. Many important new research topics emerged this year, attracting more authors’ submissions. In addition, academia’s focus and hot issues changed during this period, leading to adjustments in research directions and changes in contributing journals. Second, this study considers the impact of the introduction of new journals on the number of publications. New academic journals employ strategies to attract authors in their early days, offering a wider range of topics, faster review cycles, or higher impact factors. These factors led to more authors choosing to publish in these new journals, affecting the number of publications in 2010.

Statistical analysis is conducted on 3187 articles published by 601 authors, and the minimum number of publications for high-yield authors is calculated using Eq (8).


$$M=0.749\times \frac{1}{2}Nmax$$ (8)

Among them, aerobics, mental health, map visualization, and stress level are the highest number of publications by authors in the field. After calculation, the high-yield authors and a specific number of publications were obtained. When M ≥ 2 articles, they are classified as high-yield. The results reveal a total of 202 high-yield authors. The statistical results of the top 20 authors’ number of publications s and their respective regions are represented in Table 4. The "Regions" row in Table 4 is information about the author’s region. "Regions" here indicate the geographic region where the author’s work or academic institution is located. Each author’s region is listed in the table with its corresponding number and the number of articles published. These regions include the Southeast region, the Southwest region, the Central region, the Northeast region, and the Northwest region, etc. These regions define the different geographical locations of authors and are important for understanding the distribution of authors and the differences between regions. The regional division in Table 4 is based on the administrative regional division standard of the National Bureau of Statistics of China (except Hong Kong, Macao, and Taiwan). Specifically, the Northeast region includes Liaoning Province, Jilin Province, Heilongjiang Province; The Southeast region involves Guangdong Province, Hainan Province, Fujian Province, Zhejiang Province, Jiangxi Province, Jiangsu Province, Shanghai; The Central region covers Shanxi Province, Henan Province, Anhui Province, Hubei Province, Hunan Province, Hebei Province, Beijing City, Tianjin City, Shandong Province; The Northwest region includes Xinjiang Uygur Autonomous Region, Ningxia Hui Autonomous Region, Inner Mongolia Autonomous Region, Gansu, Shaanxi, and Qinghai provinces; The Southwest region encompasses Chongqing Municipality, Sichuan Province, Guizhou Province, Yunnan Province, Tibet Autonomous Region, and Guangxi Zhuang Autonomous Region.

**Table 4:** Authors’ number of publications and their respective regions.

**Authors’ sequence number**	**1**	**2**	**3**	**4**	**5**	**6**	**7**	**8**	**9**	**10**
**Number of publications (articles)**	7	7	6	5	4	4	4	4	4	4
**Regions**	Southeast region	Southeast region	Southwest region	Southeast region	Southeast region	Southeast region	Southeast region	Northeast region	Northeast region	Southeast region
**Authors’ sequence number**	11	12	13	14	15	16	17	18	19	20
**Number of publications (articles)**	4	4	3	3	3	3	3	3	3	3
**Regions**	Southeast region	Central region	Southwest region	Central region	Central region	Central region	Central region	Northwest region	Central region	Southeast region

Table 4 suggests that high-yield authors are mainly distributed in the Southeast region, followed by the Central, Northeast, and Northwest regions. The Southeast region is an economically developed region, so it can be seen that authors in economically developed regions have conducted more research on the impact of aerosols on mental health and stress levels.

**Fig 4:**
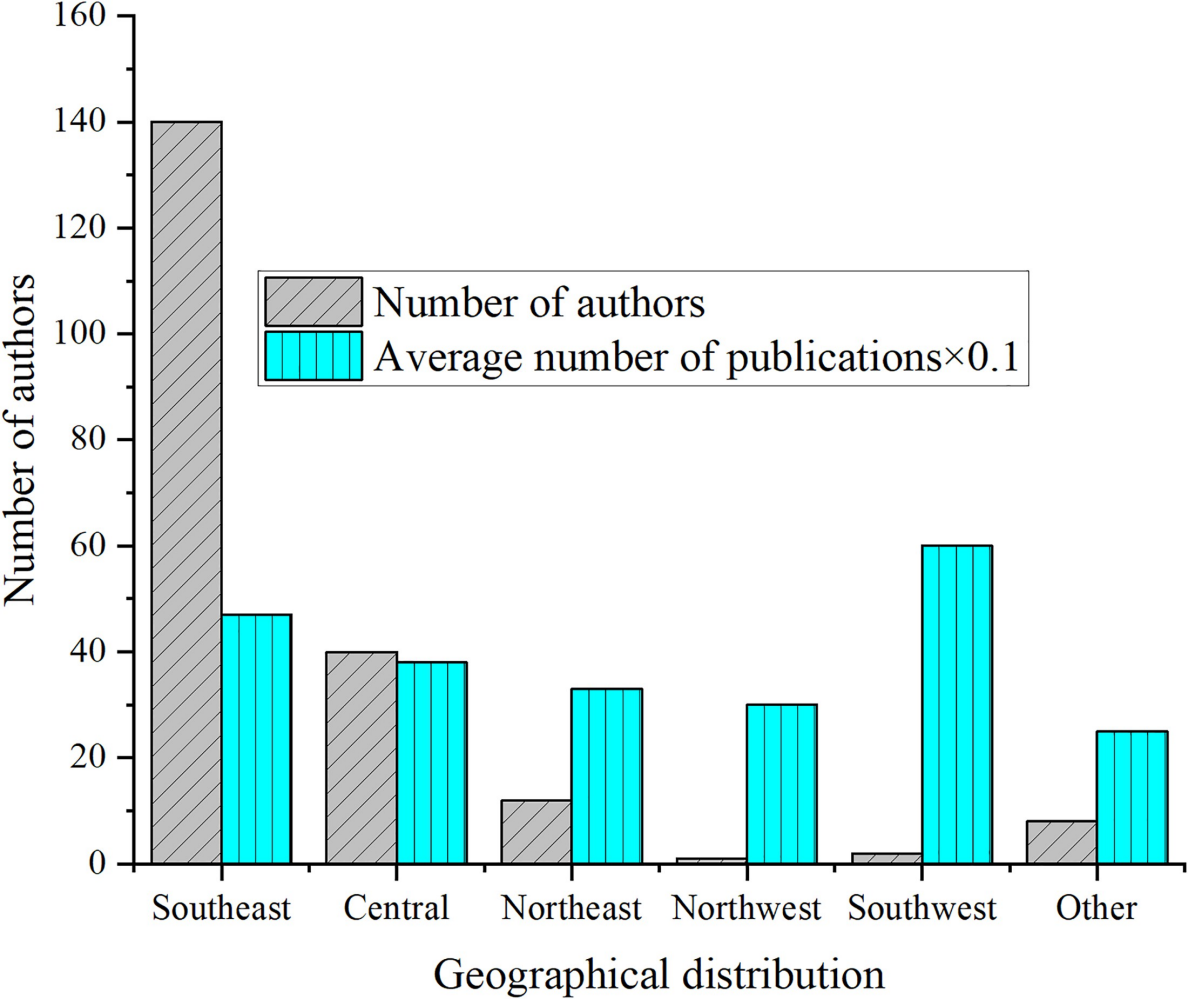
Number of publications and regional distribution of high-yield authors.

For 202 high-yield authors, statistics on some publications and regional distribution are conducted, and the results are detailed in Fig 4. For better results in Fig 4, the average number of articles in Fig 4 has been multiplied by 0.1.

Fig 4 shows that 202 high-yield authors are basically distributed in the Southeast region, supporting the conclusion of Table 4.

**Fig 5:**
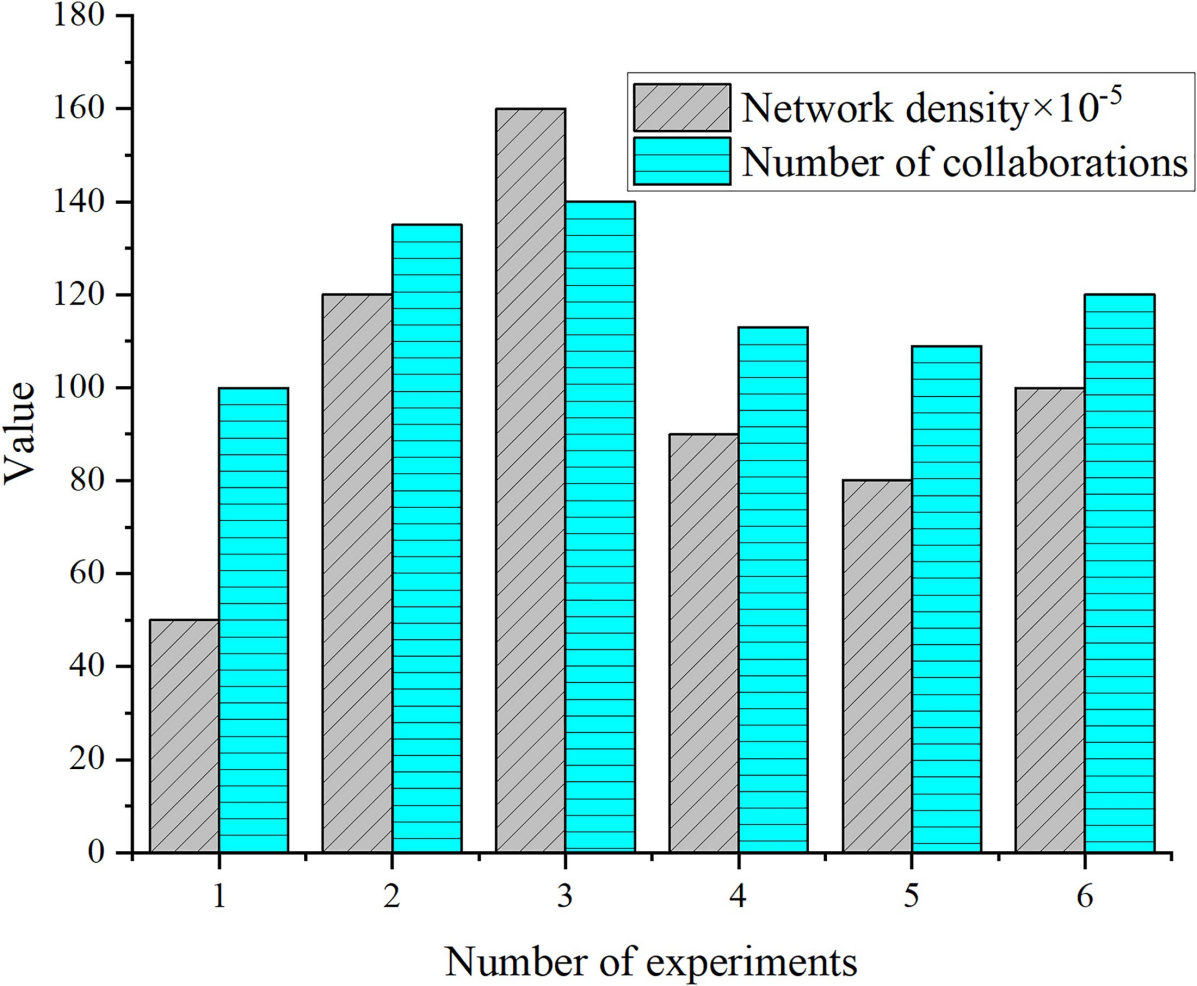
Comparison of accuracy and training time with different data amounts.

In the statistics of the co-occurrence map of authors, a total of 3,187 articles are included, involving 601 authors. A total of 6 experiments are conducted for the degree of collaboration connection between authors, and the final results of collaboration frequency and network density are drawn in Fig 5.

From the data in Fig 5, it can be observed that there are certain changes in the network density and number of collaborations in the experiment. Network density can reflect the degree of collaboration between authors, while the number of collaborations indicates their specific frequency of collaboration. Due to a network density of 0.001, the collaboration between authors is not close enough.

### DA results based on existing studies

DA was conducted based on relevant research literature that had been rigorously screened and evaluated. This research literature includes a wealth of information on the effects of aerobics on mental health and stress levels, ensuring that the analysis has a solid foundation. The existing research data are re-examined through keyword cluster analysis, author-publication analysis, and subjective feeling data collection and analysis. Keyword cluster analysis reveals the internal relationship between keywords in the literature, author-publication analysis can understand the distribution of high-yield authors and their regions, and the subjective feeling data collection provides more comprehensive support for the research results. This re-analysis aims to deepen the understanding of the existing research data and validate the findings from different perspectives. By integrating keyword clustering, author analysis, and subjective feeling data, it is possible to interpret the findings of existing studies more comprehensively, thereby providing stronger support for the findings.

**Fig 6:**
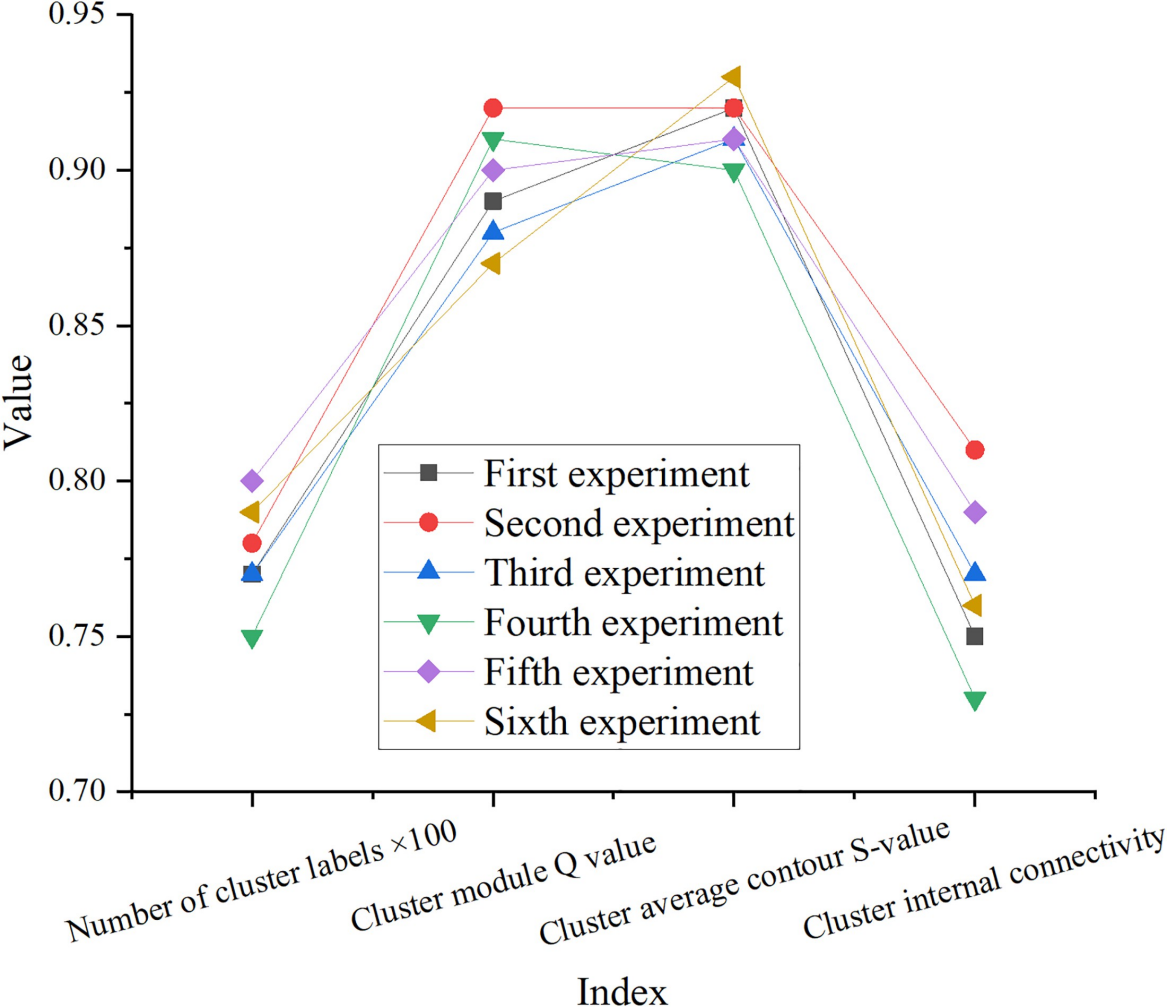
Keywords clustering results.

**Fig 7:**
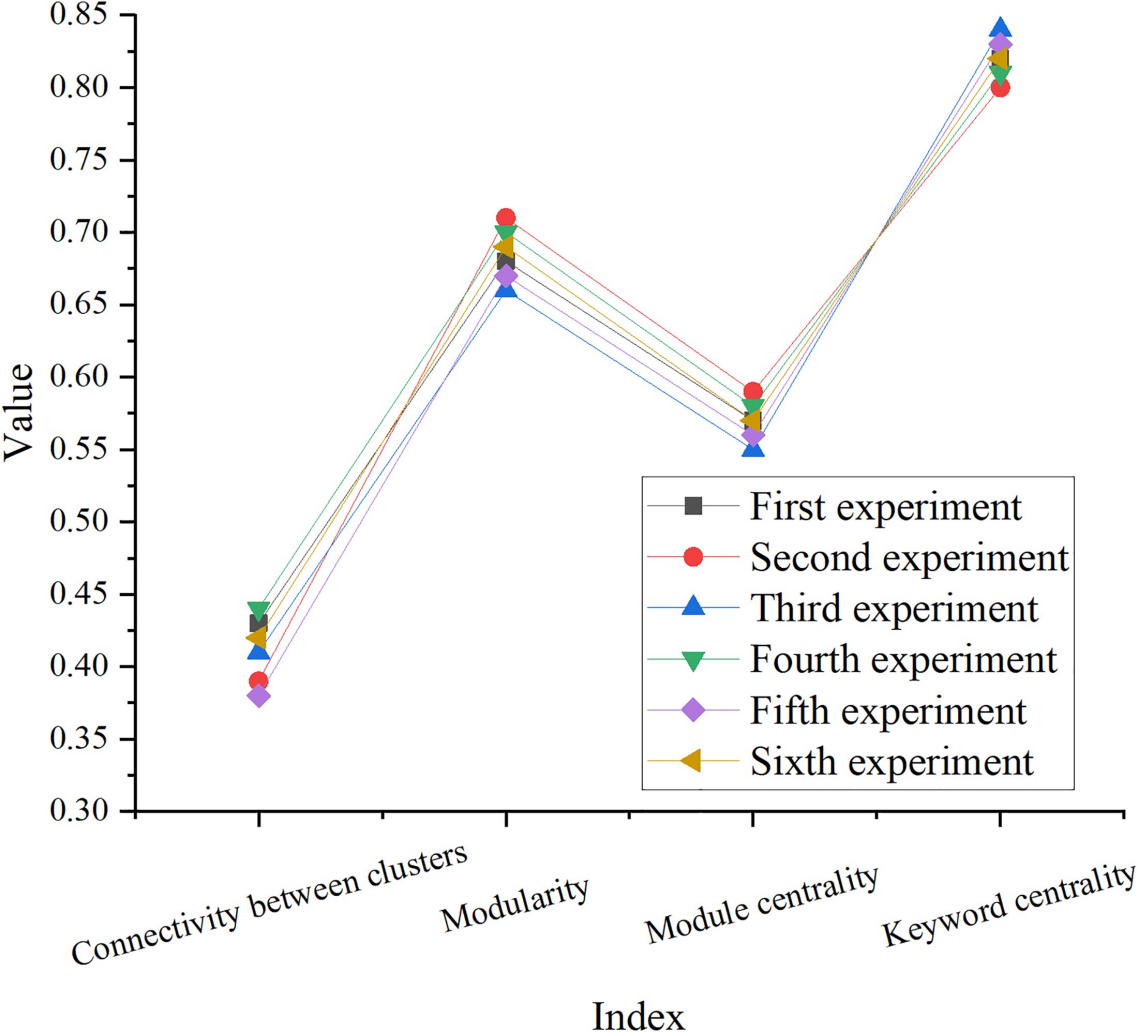
Keywords modularity results.

In the keyword cluster analysis, a total of 6 sets of experiments are conducted, and the results are suggested in Figs 6 and 7.

In Figs 6 and 7, 77 cluster labels can be obtained in the keyword clustering results. The Q value of the clustering module is 0.89, and the average clustering profile Silhouette (S) value is 0.92, illustrating that the clustering structure is prominent and the result is reasonable. According to the DA of the table, the quality of the clustering results is high, the cluster structure is significant, and the connection between different clusters is weak. Moreover, the value of modularity and module centrality is high, and the keywords have a high degree of centrality. These results signify that cluster analysis is effective for characterizing keyword clusters and network structure, and can provide useful information about the roles and characteristics of keywords and groups in the network.

**Fig 8:**
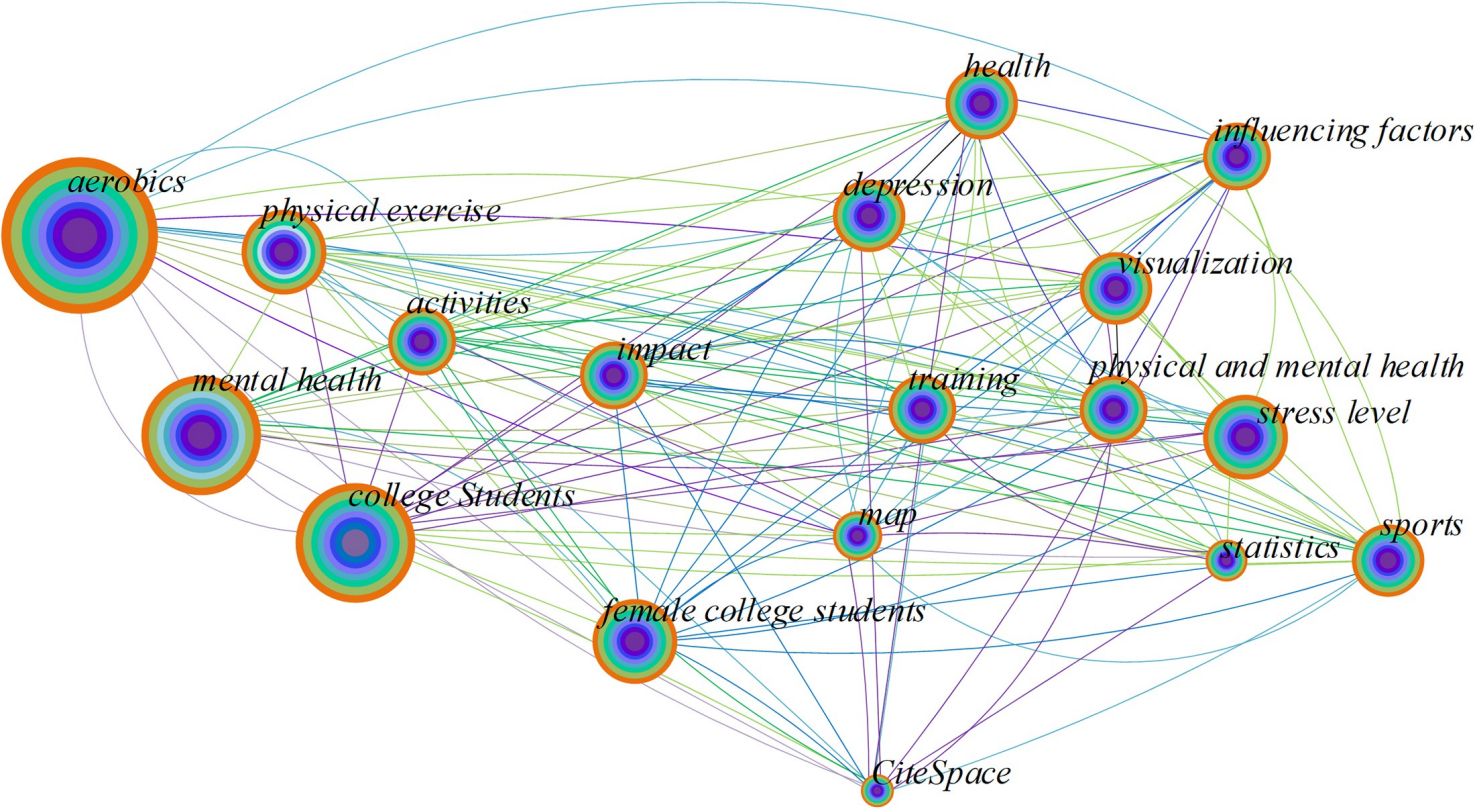
Collinear graph of keywords.

The top 20 high-frequency keywords in the field of the effect of aerobics on mental health and stress levels are suggested in Fig 8 and Table 5.

**Table 5:** The top 20 high-frequency keywords.

Keywords	Aerobics	Mental health	College Students	Physical exercise	Female college students	Stress level	Impact	Physical and mental health	Sports
**Frequency**	1,101	892	779	453	398	299	202	200	188
**Keywords**	Health	Influencing factors	Visualization	Depression	Training	Activities	Map	Statistics	CiteSpace
**Frequency**	186	176	165	159	155	140	90	76	54

In Fig 8 and Table 5, the keyword "aerobics" appeared most frequently in the study, with 1101 times, followed by "mental health" and "college students" with 892 and 779 times, respectively. This indicates that researchers are highly concerned about the influence of aerobics on mental health and stress levels, and the focus is mainly on "aerobics" and "mental health". "Aerobics" and "mental health" are two high-frequency keywords, meaning the researchers explore the positive effects of aerobics on mental health. At the same time, "stress level" and "impact" are also high-frequency keywords, indicating that the research focuses on how stress levels are affected by aerobics. The frequency of the keyword "aerobics" was relatively low before 2010, but increased significantly after 2010, which means that research on its impact has gradually increased in recent years. This may be related to the increasing popularity and awareness of aerobics. "Physical exercise" and "impact" mean the researchers focus on the potential impact of aerobics on physical exercise habits and mental states. At the same time, "visualization" and "statistics" focus on research methods and DA.

**Fig 9:**
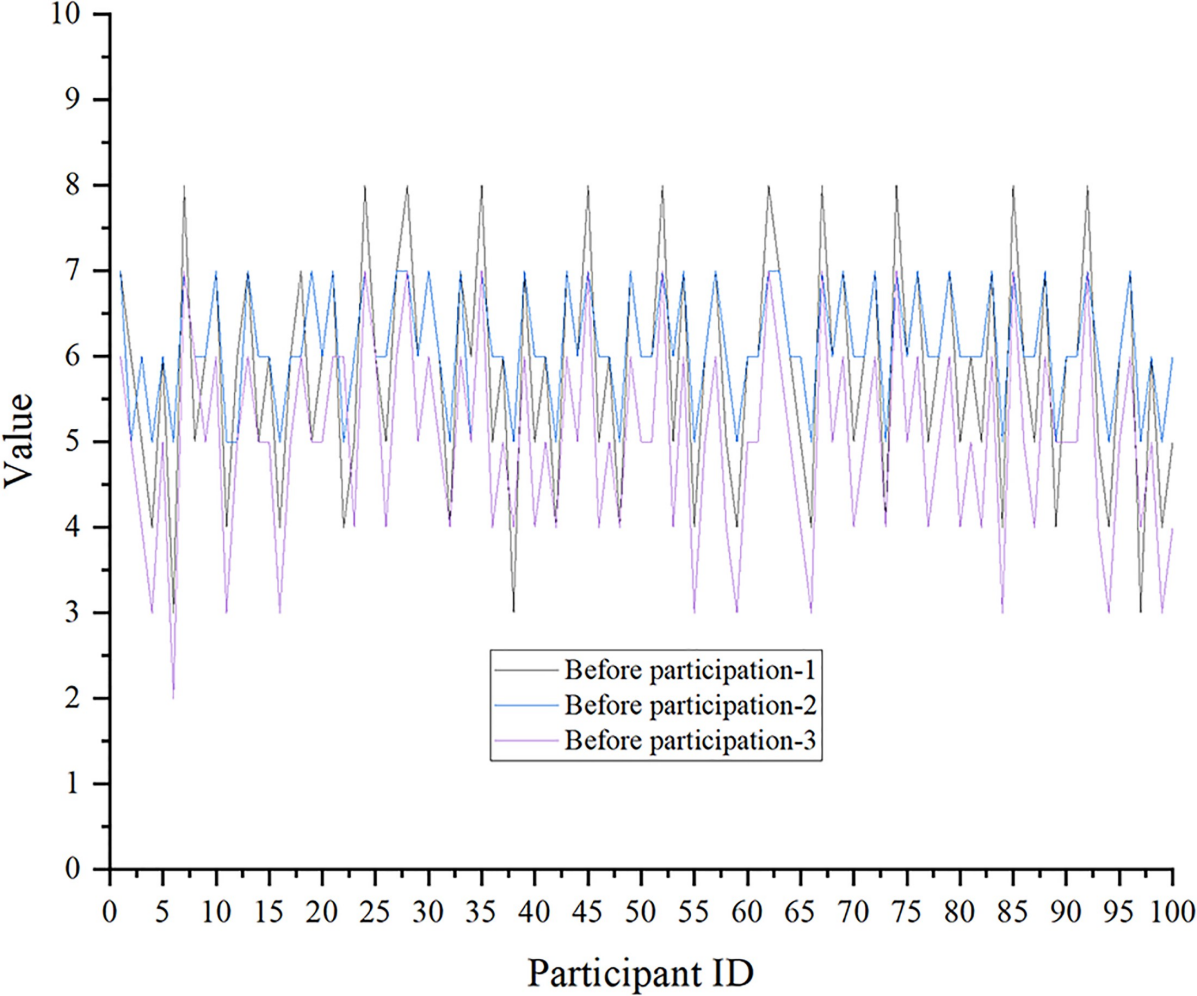
Effects of aerobics on mental health-no aerobics exercises.

**Fig 10:**
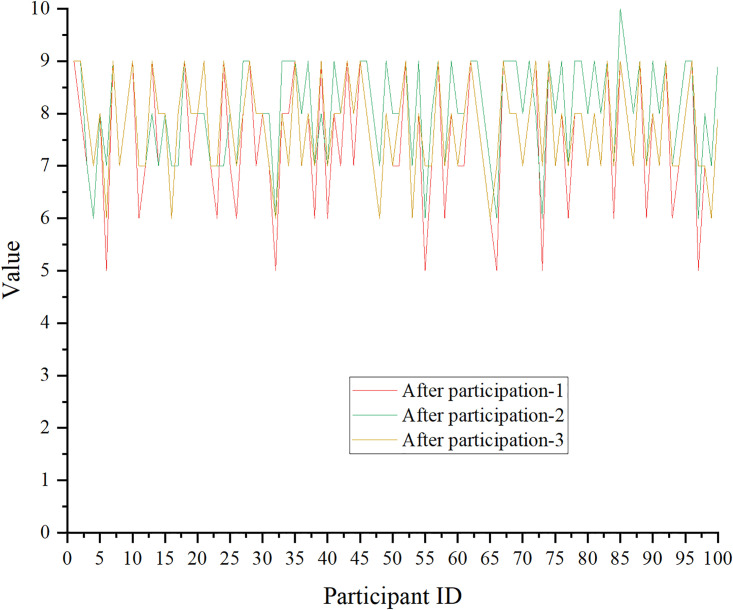
Effects of aerobics on mental health-after aerobics exercise.

According to the analysis of three groups of representative data collected in existing studies, the impact of aerobics on mental health and stress levels is portrayed in Figs 9 and 10. The score is on a 10-point scale. The mental health Inventory (MHI) is used in this study.

The score data of MHI after the aerobics exercise are drawn in Fig 9.

In Figs 9 and 10, the mental health scores of the participants are improved after the aerobics exercise. The sample t-test and significance are calculated using Excel software, and the paired sample t-test results show that the mean value of the sample mean difference is 1.61, t(100) = 4.32, p<0.05. This suggests significant differences in the pre- and post-test of individuals participating in aerobics in terms of MHI scores. Individuals participating in aerobics reveal prominent improvements in MHI scores.

**Fig 11:**
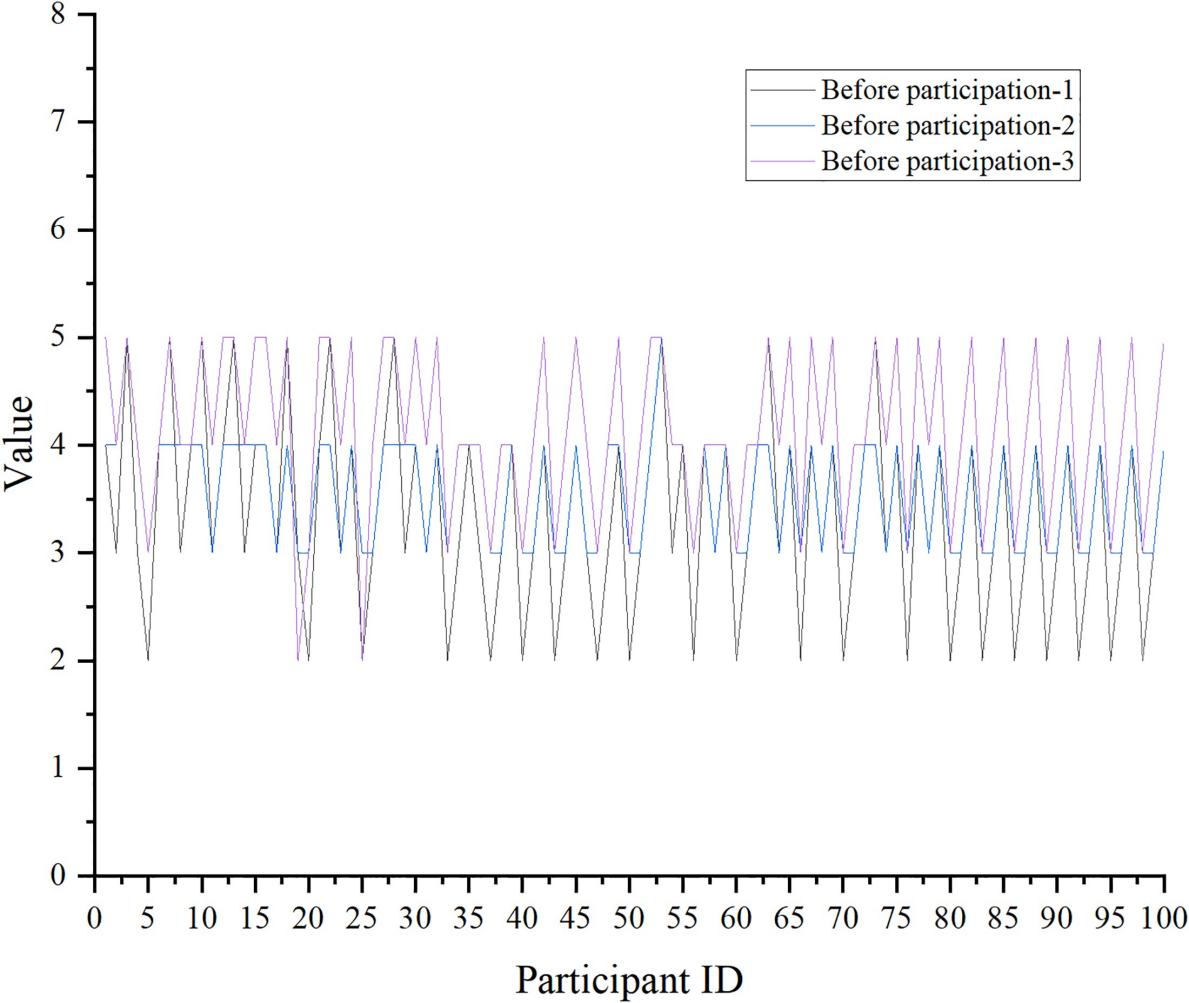
The impact of aerobics on stress levels-no aerobics exercises.

**Fig 12:**
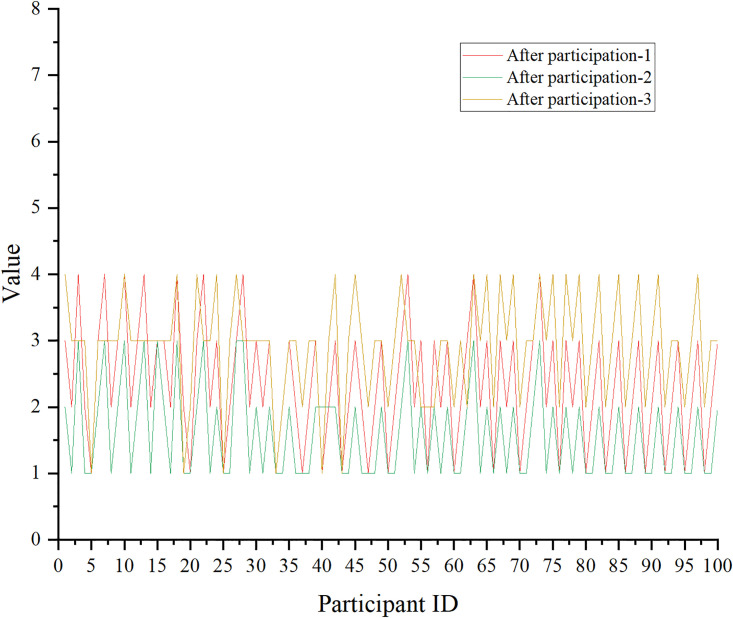
The impact of aerobics on stress levels-after aerobics exercise.

Similarly, the impact of aerobics on the pressure level is manifested in Figs 11 and 12, and the score of the pressure level is a five-point scale. The scale involved is the Perceived Stress Scale (PSS).

Fig 12 suggests the score data of PSS after aerobics exercise.

Figs 11 and 12 indicate that individuals participating in aerobics show a marked reduction in the assessment of stress level questionnaires. The results of the paired sample t-test combined with Excel reveal that the mean value of the sample mean difference is negative, t(100) = -3.91, p<0.05. This means that there is a significant difference in the assessment of stress levels among individuals participating in aerobics before and after the test, and there is a decreasing trend.

In addition, the subjective feelings of aerobics participants on mental health and stress levels in existing studies are also collected. The feelings of five representative participants collected are signified in Table 6.

**Table 6:** Subjective feelings of participants.

Participants	Subjective feelings
**A**	Aerobics really makes me feel happy and relaxed. After every practice, I feel my stress is released, and my mental state becomes calmer and more stable.
**B**	Aerobics has become one of the best ways for me to relieve stress. Every time I participate in training, I am able to focus on my movements and breathing, forgetting all troubles and stress, which greatly improves my mood.
**C**	I have been feeling anxious and nervous before, but by participating in aerobics, I have significantly decreased my stress level. I can better handle stressful situations in life and feel more confident and positive.
**D**	Aerobics has had a huge impact on my mental health. I no longer feel discouraged and depressed, but have a vibrant and optimistic attitude. I find myself more motivated to face challenges in life and able to better manage my emotions.
**E**	Participating in aerobics has notably improved my mental health and stress levels. In the past, I often felt too stressed, anxious, and difficult to relax. But since I started participating in aerobics, I have gradually felt the change.

In Table 6, participants generally believe that aerobics positively impacts their mental health and stress levels.

## Discussion

Firstly, based on the comprehensive literature analysis, from 2000 to 2009, the annual number of publications in related fields showed a notable growth trend. This reflects a significant interest among scholars in topics such as aerobics, mental health, stress levels, and graph visualization during this period. Secondly, it is worth noting that between 2010 and 2017, the number of publications fluctuated, reflecting the fluctuation of scholars’ attention to this field during this period. This fluctuation is due to factors such as changes in academic hotspots and the emergence of new research directions. Finally, from 2018 to 2022, the number of publications increased, indicating that the field has maintained a stable level of research activity in recent years. The increasing health awareness and the growing attention to mental health issues influence this. The analysis of the regional distribution of high-yield authors shows that high-yield authors are mainly distributed in the Southeast region, followed by the Central, Northeast, and Northwest regions. This can be interpreted as economically developed regions having the conditions and resources to conduct relevant research more easily. As an economically developed region, the Southeast region has many high-yield authors, which may reflect their investment in supporting mental health and exercise research. This also suggests that attention can be paid to non-high-yield regions in future research to obtain a more comprehensive research perspective. Through keyword clustering and thematic analysis, it can be found that aerobics and mental health are the most frequent keywords. This means that scholars are paying more attention to the relationship between aerobics and mental health in their research. Especially, the keyword clustering results show a close correlation among aerobics, mental health, and stress levels. This indicates that scholars tend to comprehensively consider the role of aerobics in improving mental health and reducing stress levels in their research. The scores of the mental health assessment and stress level questionnaire both present a remarkable improvement in participants’ mental health and stress levels after aerobics exercise. This is consistent with the participants’ subjective feelings, further strengthening the view that aerobics positively affects mental health. In future research, in addition to objective data, in-depth exploration of individual subjective feelings is also crucial. However, there are some shortcomings. First, this study focuses on Chinese Mainland, and more research is needed on the impact of other cultural backgrounds. Second participants’ mental health and stress levels, the period that this study focuses on is relatively short, and it is not possible to fully understand the impact of long-term participation in aerobics on mental health. Future research can consider adopting more long-term observations and cross-cultural research designs. Lastly, the coverage of the literature is also limited, and some related studies may not have been included in the analysis. Future research can further expand the scope of literature to obtain more comprehensive research conclusions.

## Conclusions

This study explores the impact of aerobics on individual mental health and stress levels through scale evaluation and subjective feedback from participants. The overall number of literatures on aerobics, mental health, stress levels, and graph visualization is rising, with an average annual publication of around 190 articles. High-yield authors are mainly distributed in the Southeast region, followed by the Central, Northeast, and Northwest regions. In the keyword clustering results, a total of 77 cluster labels are obtained, with a Q value of 0.89 for the clustering module and an average contour S value of 0.92, illustrating significant clustering structure and reasonable results. After participating in aerobics exercise, the psychological health scores of the participants are all improved, with an average difference of 1.61 in sample mean, t(100) = 4.32, p<0.05. Individuals participating in aerobics also show a marked decrease in stress level questionnaire evaluation. Based on the research results, the following conclusions can be drawn. Firstly, participating in aerobics significantly improves an individual’s mental health level. Participants exhibit better emotional regulation ability and mental health status in the MHI evaluation. This result supports the positive impact of aerobics on individual mental health. Secondly, participating in aerobics can effectively reduce an individual’s stress level. Participants have a prominent decrease in stress level assessment, experiencing physical and mental relaxation and calmness. This indicates that aerobics has a positive effect on stress management. However, there are some limitations to this study. The relatively small sample size limits the generalization ability of the results. This study focuses on a relatively short period, and it is impossible to comprehensively understand the impact of long-term participation in aerobics on mental health. Future exploration can expand sample size, increase research reliability and representativeness, and adopt more long-term observation and cross-cultural research designs. In addition, this study does not involve the measurement of physiological indicators, and there is still room for improvement in the coverage of literature descriptions. Future research can combine physiological measurements to further explore the impact of aerobics on individuals and add more descriptions of literature and more presentation of image results.

## Supporting information

S1 Data (XLSX)
